# Outcomes of experimental infection of calves with swine influenza H3N2 virus

**DOI:** 10.1128/mbio.03957-24

**Published:** 2025-06-12

**Authors:** Lei Shi, Yuekun Lang, Sawrab Roy, Zhenyu Shen, Dipali Gupta, Chao Dai, Muhammad Afnan Khalid, William J. Mitchell, Shuping Zhang, Richard Webby, Juergen Richt, Wenjun Ma

**Affiliations:** 1 Department of Veterinary Pathobiology, College of Veterinary Medicine, University of Missouri, Columbia, Missouri, USA; 2 Department of Molecular Microbiology & Immunology, School of Medicine, University of Missouri, Columbia, Missouri, USA; 3 MU Center for Influenza and Emerging Infectious Diseases, University of Missouri 14716, Columbia, Missouri, USA; 4 Veterinary Medical Diagnostic Laboratory, College of Veterinary Medicine, University of Missouri 14716, Columbia, Missouri, USA; 5 Department of Host-Microbe Interactions, St Jude Children's Research Hospital 5417, Memphis, Tennessee, USA; 6 Department of Diagnostic Medicine/Pathobiology, College of Veterinary Medicine, Kansas State University 5308, Manhattan, Kansas, USA; Icahn School of Medicine at Mount Sinai, New York, New York, USA

**Keywords:** cattle respiratory and mammary gland cells, calves, swine and avian influenza viruses, infection, transmission

## Abstract

Unprecedented outbreaks caused by the H5N1 highly pathogenic avian influenza virus (HPAIV) among dairy cows in the United States have raised significant concerns. Whether other subtypes of influenza A viruses (IAVs) can infect and transmit in cattle remains largely unknown. Herein, we infected cattle respiratory and mammary gland cells with different IAVs and two groups of Holstein calves intranasally or orally with a swine H3N2 virus to determine their susceptibility. Naive calves were co-housed with infected animals to investigate virus transmission. Results showed that tested swine and avian IAVs could infect cattle primary nasal turbinate and tracheal epithelial cells, as well as immortalized mammary gland epithelial cells and fibroblasts. No obvious clinical signs, including fever, were observed in infected and contact calves, but macroscopic lung lesions were found in necropsied animals in both groups on day 5 post-infection. Viral shedding was detected in three out of four nasally infected calves but not in orally infected or the two groups of contact animals. Interestingly, viral RNA and antigen could not be detected in all tissues from individual necropsied animals from either infection group, but viral RNA and sequences were detected in serum samples of two nasally infected calves on day 7 post-infection, not on other days and in other animals. Additionally, only the nasally infected animals seroconverted. Our results indicate that in addition to H5N1 HPAIV, swine H3N2 virus can infect cattle but does not transmit efficiently among them, suggesting that other subtypes of IAVs could infect and replicate in cattle.

**IMPORTANCE:**

Highly pathogenic avian influenza H5N1 virus outbreaks in U.S. dairy herds have raised questions about whether other subtypes of influenza A viruses (IAVs) can infect and transmit in cattle. In this study, we investigated the susceptibility and infection of different IAVs in bovine primary and immortalized cells and Holstein calves. Results showed that avian H5N1 and H9N2, and swine H3N2 IAVs could infect beef cattle primary nasal turbinate and tracheal epithelial cells, as well as immortalized mammary gland epithelial cells and fibroblasts. Moreover, the swine H3N2 could infect the calves through intranasal infection, but not through oral infection, despite no obvious clinical signs and efficient transmission being observed. Our results demonstrate that other subtypes of IAVs can infect cattle and might pose threats to public and animal health.

## INTRODUCTION

Influenza A virus (IAV) is an important zoonotic pathogen that can infect birds and mammalian species, including humans. IAV is responsible for human seasonal epidemics and occasional pandemics. In the United States, approximately 50,000 people die annually from seasonal influenza, and influenza pandemics can result in millions of deaths worldwide. Pandemics typically arise from the introduction of novel reassortant avian or animal IAVs into humans, as people lack preexisting immunity to these novel strains. Historically, bovines were not considered natural hosts or susceptible species due to the presence of certain anti-influenza host factors (1). Limited serological evidence has suggested sporadic IAV infections in cattle; however, few virus strains have been isolated despite occasional influenza-like disease in some herds (1). In March 2024, an unprecedented H5N1 highly pathogenic avian influenza virus (HPAIV) clade 2.3.4.4b genotype B3.13 spillover occurred in Texas dairy cows and was subsequently detected in multiple dairy herds across several states (2–4). As of 16 December 2024, 860 dairy herds have been affected across 16 U.S. states (5), and 60 human infection cases were confirmed, with nearly all linked to direct contact with HPAIV H5N1-affected cattle or poultry farms (6). Meanwhile, cattle-origin HPAIV H5N1 has been reported to transmit to several domestic and peridomestic mammalian animals as well as poultry species (3). These developments highlight bovines’ critical role in the interspecies transmission of the current HPAIV H5N1 outbreak, raising significant concerns for public and animal health.

The limited number of surveillance studies conducted has revealed that bovine serum samples predominantly tested positive for human H1N1 and H3N2 IAVs despite the presence of swine virus antibodies (1). In the early 1970s, antibodies against the human H3N2 virus were detected in cattle and yaks in India and Nepal (7). A seroprevalence study showed that 1.5% and 1% of 728 cattle serum samples collected from 1975 to 1977 in Japan tested positive against H1 and H3, respectively (8). Two studies conducted in the United Kingdom showed that more than 50% of cattle serum samples were seropositive against human H1N1 and/or H3N2 IAVs (9, 10). Interestingly, another serological study including 177 calf serum samples collected from 1978 to 1981 in the United States found that 3.4% of the calves were seropositive to swine H1N1 virus (11). A separate seroprevalence study reported that 51% of cattle sera collected in Kentucky between 1999 and 2000 were seropositive for swine H3N2 virus, and 17% tested positive for equine H3N8 virus (12). This cumulative serological evidence indicates that bovine species of various ages can be infected by different IAVs. Depending on subtype, the IAVs induced variable clinical signs in cattle. Experimental intranasal inoculation of a swine H1N1 IAV in calves caused respiratory disease with virus shedding, contact transmission to sentinel animals, and seroconversion (13). A bovine H3N2 isolate could cause an influenza-like illness and be detected for 7 days in infected calves, while three human H3N2 viruses did not induce disease in infected calves (14). Furthermore, calves experimentally infected with an HPAIV H5N1 virus exhibited moderate viral shedding, seroconversion, and contact transmission (15). In contrast, inoculation of calves with the equine H3N8 virus did not result in any clinical symptoms, viral shedding, or seroconversion (12). Studies using the U.S. bovine H5N1 B3.13 virus showed that it replicated in the udder of cows when delivered by the intramammary route. Moderate nasal replication and shedding with no severe clinical signs were observed in intranasally inoculated calves or heifers inoculated by an aerosol respiratory route (16, 17). As IAVs evolve rapidly, infection of bovines could generate mammalian-adapted viruses with increased human infection risks. Considering the rapid spreading in cattle herds and interspecies transmission of H5N1 HPAIVs, urgent systemic surveillance of IAV infections in bovine herds and experimental cattle infection studies with different subtypes of IAVs are crucial to understand their susceptibility, pathogenicity, and transmission, as well as reassortment potential.

Here, we experimentally infected cattle respiratory and mammary gland cells with avian and swine IAVs, and calves with a swine H3N2 virus through the nasal and oral routes. Our results demonstrated that cattle primary respiratory epithelial cells, immortalized mammary gland epithelial cells, and fibroblasts were susceptible to infection with tested swine H3N2 and avian H5N1 and H9N2 IAVs, and the swine H3N2 virus could infect calves but did not replicate extensively and did not transmit to sentinel animals.

## RESULTS

### Susceptibility of bovine primary respiratory and immortalized mammary gland cells to infection with IAVs

**Fig 1:**
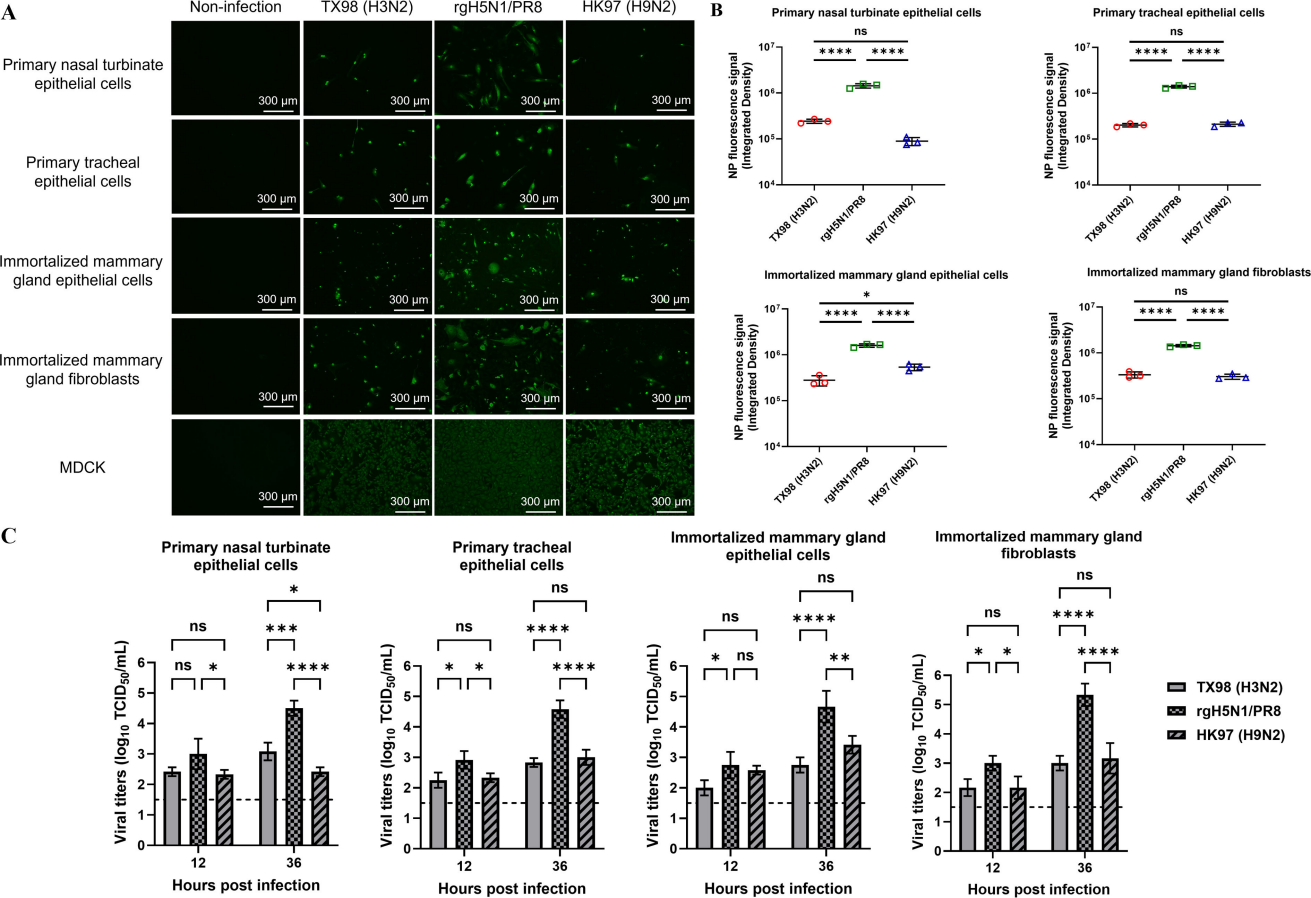
Bovine primary respiratory and immortalized mammary gland cells infected with different swine and avian IAVs. (A) Bovine primary nasal turbinate and tracheal epithelial cells, as well as immortalized mammary gland epithelial cells and fibroblasts, were infected with swine H3N2, avian rgH5N1/PR8, or avian H9N2 viruses at a multiplicity of infection (MOI) of 1. At 24 h post-infection, cells were fixed and stained with an anti-IAV NP monoclonal antibody. (B) Mean values of the integrated fluorescence density from three separate biological repeats were compared among bovine cells infected with different IAVs. The result was analyzed by using a one-way ANOVA followed by Tukey’s multiple comparison. (C) Four bovine cells were infected with swine H3N2, avian rgH5N1/PR8, or avian H9N2 viruses at an MOI of 0.1. The supernatants were collected at 12 and 36 h post-infection and were titrated on MDCK cells. The result was analyzed by using a two-way ANOVA (*P < 0.05, **P < 0.01, ***P < 0.001, and ****P < 0.0001).

**Fig 2:**
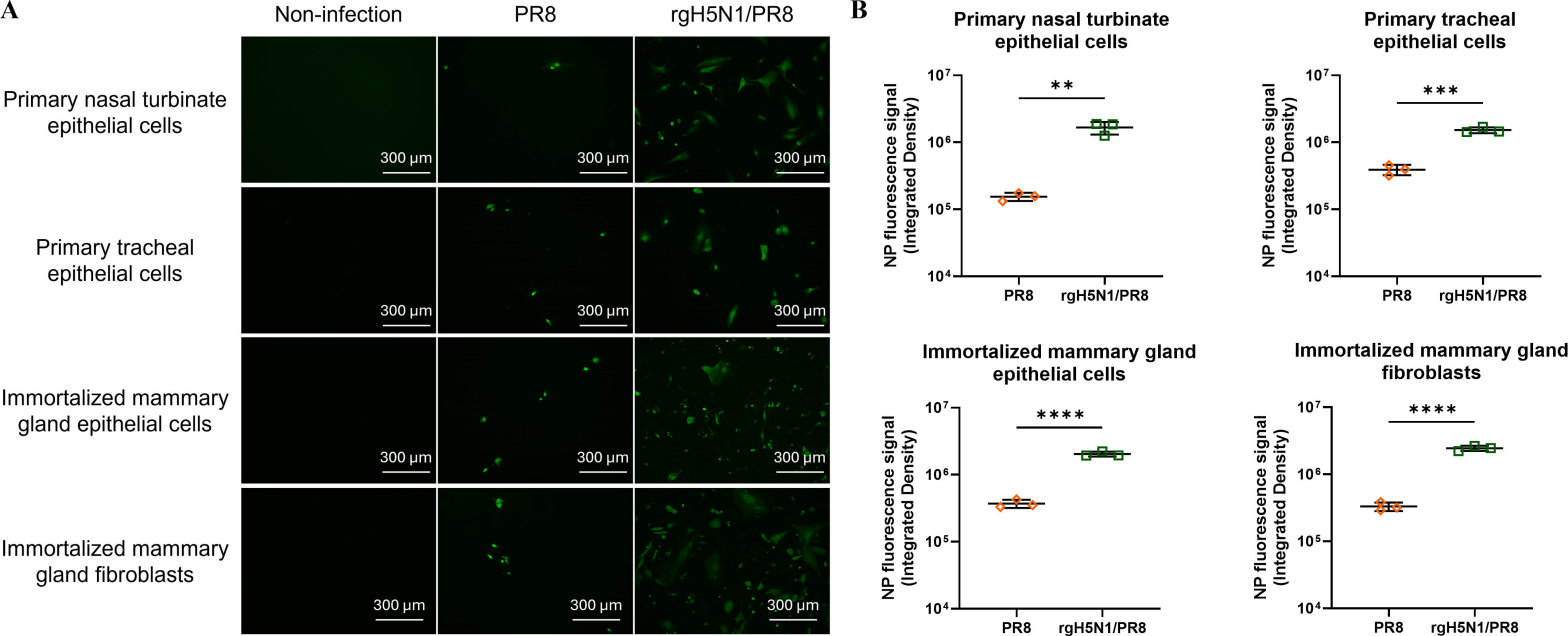
Bovine primary respiratory and immortalized mammary gland cells infected with PR8 and rgH5N1/PR8. (A) Bovine primary nasal turbinate and tracheal epithelial cells, as well as immortalized mammary gland epithelial cells and fibroblasts, were infected with the PR8 or rgH5N1/PR8 at a multiplicity of infection of 1. At 24 h post-infection, cells were fixed and stained with an anti-IAV NP monoclonal antibody. (B) Mean values of integrated fluorescence density from three separate biological repeats were compared in each bovine cell line infected with PR8 or rgH5N1/PR8. The result was analyzed by using a Student’s *t* test (**P < 0.01, ***P < 0.001, and ****P < 0.0001).

To investigate the susceptibility of bovine respiratory and mammary gland cells to infection with different IAVs, we produced and established primary beef cattle nasal turbinate and tracheal epithelial cells, as well as mammary gland epithelial cells and fibroblasts. Furthermore, we immortalized mammary gland epithelial cells and fibroblasts (Fig. S1A) and validated these bovine epithelial cells and fibroblasts by staining them with specific anti-cytokeratin and anti-smooth muscle actin (SMA) antibodies as shown in Fig. S1B. Primary cattle nasal turbinate and tracheal epithelial cells, as well as immortalized mammary gland epithelial cells and fibroblasts, were infected with the avian rgH5N1/PR8, avian HK97 H9N2, or swine TX98 H3N2 viruses. Results revealed that primary cattle nasal turbinate and tracheal epithelial cells, as well as immortalized mammary gland epithelial cells and fibroblasts, were susceptible to infections with avian rgH5N1/PR8, HK97 H9N2, and swine TX98 H3N2 viruses when compared to control Madin-Darby canine kidney (MDCK) cells (Fig. 1A). Interestingly, significantly more infected cells with NP signals were detected in the rgH5N1/PR8 infected bovine cells than those found in either avian H9N2 or swine H3N2-infected respective cells (Fig. 1B). Moreover, the rgH5N1/PR8 replicated more efficiently in these four bovine cells than both avian HK97 H9N2 and swine TX98 H3N2 viruses according to virus titers (Fig. 1C), which is consistent with the results based on infected cells with NP signals. Additionally, no significant difference in virus titers was observed between swine TX98 H3N2 and avian HK97 H9N2 viruses in tested four bovine cells, except for in primary nasal turbinate epithelial cells, in which the TX98 H3N2 exhibited a higher titer than the HK97 H9N2 virus at 36 h post-infection (Fig. 1C). To determine whether six internal genes from the PR8 H1N1 virus play a major role in determining the rgH5N1/PR8 efficient infection and replication in bovine cells, we infected these primary and immortalized bovine cells with the PR8 H1N1 or rgH5N1/PR8 virus. Results showed that significantly more infected cells with NP signals were detected in rgH5N1/PR8-infected bovine cells than those seen in PR8 H1N1-infected respective cells (Fig. 2), indicating that both the HA and NA genes from the clade 2.3.4.4b H5N1 virus, not six internal genes from the PR8 H1N1 virus, are crucial for rgH5N1/PR8 effective infection and replication in bovine cells. These results indicate that bovine cells are more susceptible to the avian H5N1 virus than both swine H3N2 and avian H9N2 viruses and that different subtypes of IAVs might have distinct tissue tropism in cattle. Taken together, *in vitro* results demonstrate that bovine cells can be infected by avian and swine IAVs, but the susceptibility of the same cells to different subtypes of IAVs varies.

### Clinical signs and virus shedding in inoculated calves

**Fig 3:**
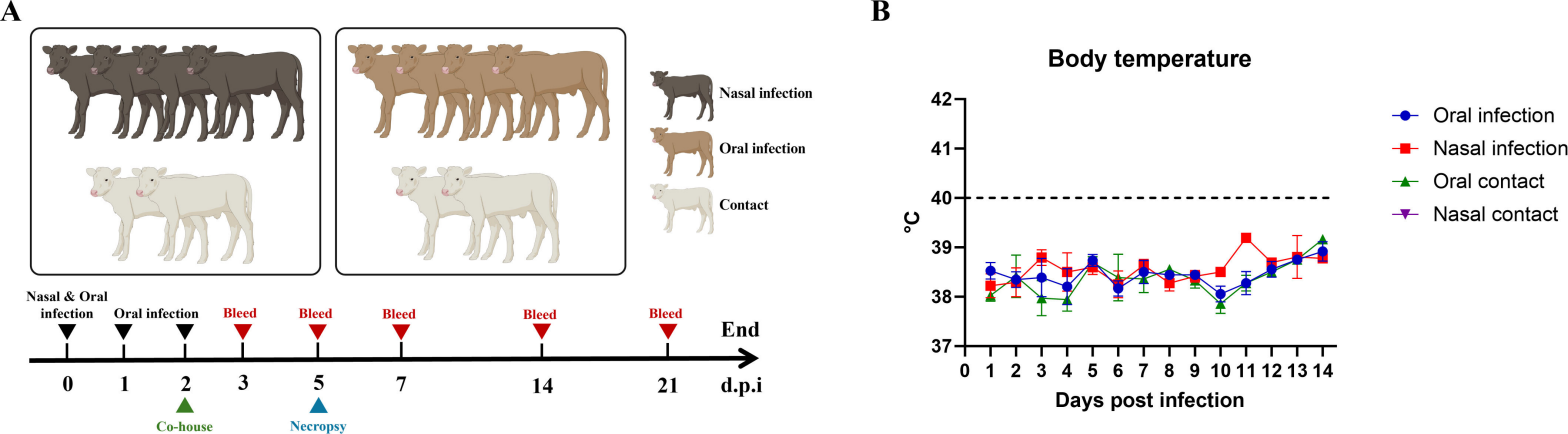
Experimental design of calf infection study and body temperature of infected and contact calves. (A) Experimental design of calf infection study. Groups of four calves were intranasally or orally inoculated with the TX98 virus, and two naive animals were co-housed with infected calves in each group at day 2 post-infection. Clinical signs were monitored daily. (B) Body temperature of infected and contact calves.

No obvious clinical signs were observed in calves inoculated through either intranasal or oral routes for 21 days post-infection (dpi). No fever was detected in any infected calf in either infection group, with body temperature ranging from 37.5°C to 39.5°C during the 14-day observation period (Fig. 3B). To determine virus shedding, nasal, oropharyngeal, and rectal swab samples were collected from each infected animal. Nasal swabs collected from three out of four calves in the intranasal infection group tested positive by RT-qPCR, while oropharyngeal and rectal swabs from all four infected animals in this group were negative. Calf #175 tested positive in nasal swabs collected on days 1, 3, and 5 post-infection, and nasal swabs collected from calf #4073 on days 1, 2, 3, and 4 post-infection were positive, while nasal swabs collected on days 1 and 4 post-infection from calf #4074 tested positive (Table 1). In contrast, all swab samples, including nasal, oropharyngeal, and rectal swabs collected from orally inoculated animals, were negative by RT-qPCR (Table 1). These results indicate that nasal infection results in more effective virus shedding than oral infection in calves.

**TABLE 1:** Results of swab samples tested by RT-qPCR^*a*^

Group	Calf		1 dpi			2 dpi			3 dpi			4 dpi			5 dpi			6 dpi			7 dpi			8 dpi			9 dpi			10 dpi			11 dpi			12 dpi		
	Type	No.	N	O	R	N	O	R	N	O	R	N	O	R	N	O	R	N	O	R	N	O	R	N	O	R	N	O	R	N	O	R	N	O	R	N	O	R
1 (nasal infection)	Infected	#175	+	−	−	−	−	−	+	−	−	−	−	−	++	−	−	N/A	N/A	N/A	N/A	N/A	N/A	N/A	N/A	N/A	N/A	N/A	N/A	N/A	N/A	N/A	N/A	N/A	N/A	N/A	N/A	N/A
		#4073	+	−	−	++	−	−	++	−	−	+	−	−	−	−	−	N/A	N/A	N/A	N/A	N/A	N/A	N/A	N/A	N/A	N/A	N/A	N/A	N/A	N/A	N/A	N/A	N/A	N/A	N/A	N/A	N/A
		#166	−	−	−	−	−	−	−	−	−	−	−	−	−	−	−	−	−	−	−	−	−	−	−	−	−	−	−	−	−	−	−	−	−	−	−	−
		#4074	+	−	−	−	−	−	−	−	−	++	−	−	−	−	−	−	−	−	−	−	−	−	−	−	−	−	−	−	−	−	−	−	−	−	−	−
	Contact	#176	−	−	−	−	−	−	−	−	−	−	−	−	−	−	−	−	−	−	−	−	−	−	−	−	−	−	−	−	−	−	−	−	−	−	−	−
		#4075	−	−	−	−	−	−	−	−	−	−	−	−	−	−	−	−	−	−	−	−	−	−	−	−	−	−	−	−	−	−	−	−	−	−	−	−
2 (oral infection)	Infected	#165	−	−	−	−	−	−	−	−	−	−	−	−	−	−	−	N/A	N/A	N/A	N/A	N/A	N/A	N/A	N/A	N/A	N/A	N/A	N/A	N/A	N/A	N/A	N/A	N/A	N/A	N/A	N/A	N/A
		#162	−	−	−	−	−	−	−	−	−	−	−	−	−	−	−	N/A	N/A	N/A	N/A	N/A	N/A	N/A	N/A	N/A	N/A	N/A	N/A	N/A	N/A	N/A	N/A	N/A	N/A	N/A	N/A	N/A
		#164	−	−	−	−	−	−	−	−	−	−	−	−	−	−	−	−	−	−	−	−	−	−	−	−	−	−	−	−	−	−	−	−	−	−	−	−
		#168	−	−	−	−	−	−	−	−	−	−	−	−	−	−	−	−	−	−	−	−	−	−	−	−	−	−	−	−	−	−	−	−	−	−	−	−
	Contact	#161	−	−	−	−	−	−	−	−	−	−	−	−	−	−	−	−	−	−	−	−	−	−	−	−	−	−	−	−	−	−	−	−	−	−	−	−
		#174	−	−	−	−	−	−	−	−	−	−	−	−	−	−	−	−	−	−	−	−	−	−	−	−	−	−	−	−	−	−	−	−	−	−	−	−

^*a*^ “−,” Ct ≥ 40; +, 35 ≤ Ct < 40; ++, 30 ≤ Ct < 35; and N/A, not collected.

### Virus replication and pathogenicity in inoculated calves

**Fig 4:**
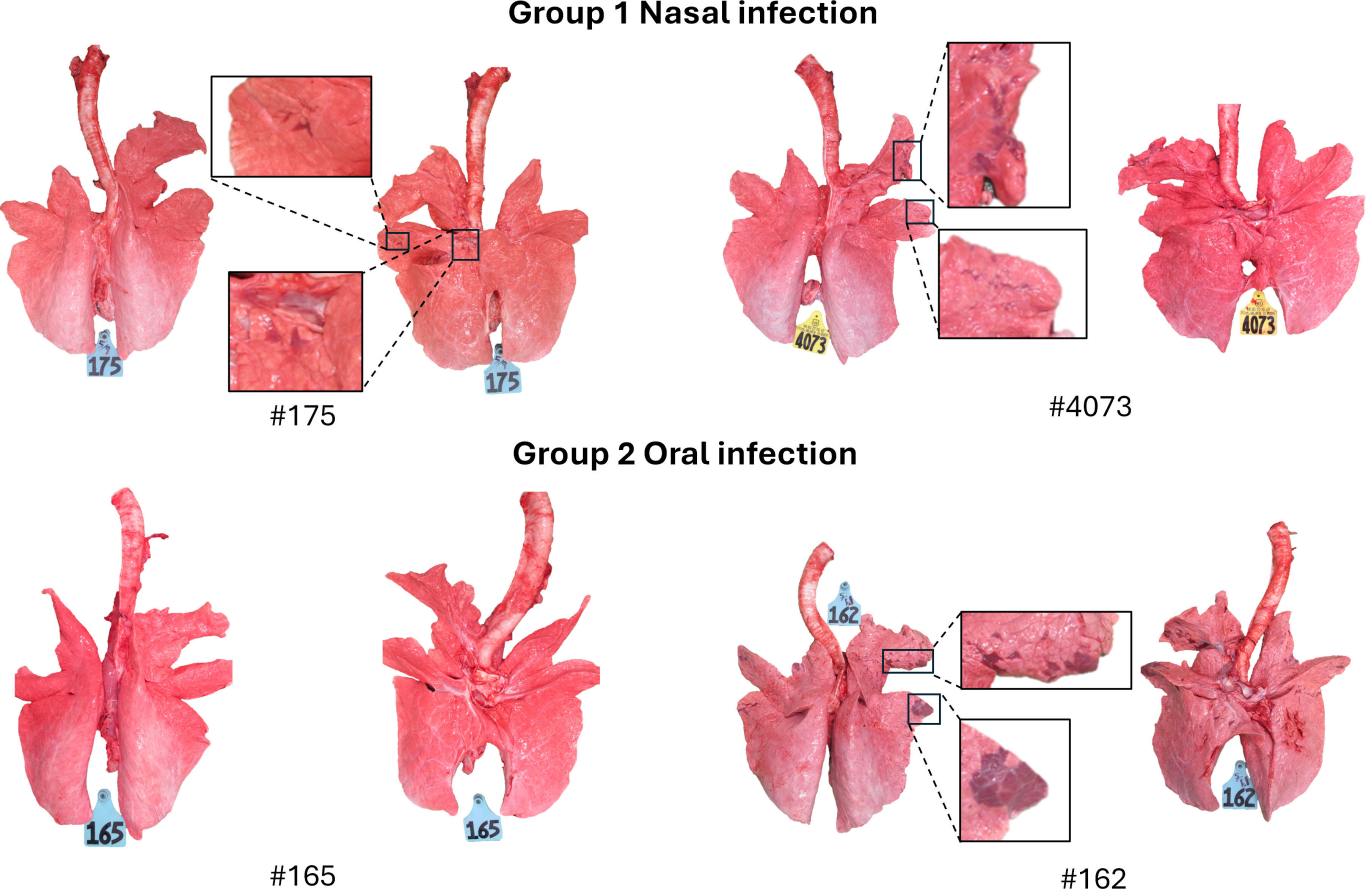
Macroscopic lung lesion of necropsied calves on day 5 post-infection. Lung lesions were observed in two intranasally infected calves (#175 and #4073) and one orally infected calf #162. The lesions were found in the right cranial and cardiac lung lobes.

RNA from all tissue samples, including the abomasum, kidney, liver, ileocecal lymph node (LN), inguinal LN, mandibular LN, mesenteric LN, parotid LN, popliteal LN, retropharyngeal LN, lung, omasum, reticulum, rumen, spleen, thymus, trachea, turbinate, brainstem, and cerebellum, from calves necropsied on day 5 post-infection from the nasal and oral infection groups tested negative by RT-qPCR assay (Table S1). Interestingly, viral RNA was only detected in serum samples from two infected calves in the nasal infection group on day 7 post-infection, but not on other days from these two animals or other animals in both groups (Table 2). Deep sequencing confirmed this finding, detecting 126 reads of the TX98 genome sequence from the sample of calf #166 and 304 reads from the sample of calf #4074 (Table S2). Three gene segments, including PB2, PA, and HA, were detected in the serum sample from calf #166 with one amino acid mutation G578A in the PA. In contrast, seven gene segments, except for NS, were found in the serum sample of calf #4074, and three amino acid mutations were identified in NP (E260Q and L270R) and NA (V66E) (Table S3). Minimal macroscopic lung lesions (purple-red consolidation) were observed in the lungs of one (#162) out of two calves (#165 and #162) in the oral infection group and the lungs of two animals (#175 and #4073) in the nasal infection group (Fig. 4). The lesions were predominantly located in the right cranial and cardiac lung lobes (Fig. 4). Correspondingly, microscopic lesions were observed in nasal turbinate, trachea, and lung tissues in necropsied calves in both infection groups (Fig. S2). However, no viral NP antigen was detected by immunohistochemistry (IHC) in these animals.

**TABLE 2:** Results of serum samples tested by RT-qPCR^*a*^

Group	Calf		3 dpi	5 dpi	7 dpi	14 dpi	21 dpi
	Type	No.					
1 (nasal infection)	Infected	#175	−	−	N/A	N/A	N/A
		#4073	−	−	N/A	N/A	N/A
		#166	−	−	+	−	−
		#4074	−	−	+	−	−
	Contact	#176	−	−	−	−	−
		#4075	−	−	−	−	−
2 (oral infection)	Infected	#165	−	−	N/A	N/A	N/A
		#162	−	−	N/A	N/A	N/A
		#164	−	−	−	−	−
		#168	−	−	−	−	−
	Contact	#161	−	−	−	−	−
		#174	−	−	−	−	−

^*a*^ “−,” Ct ≥ 40; +, Ct 35 ≤ Ct < 40; and N/A, not collected.

**Fig 5:**
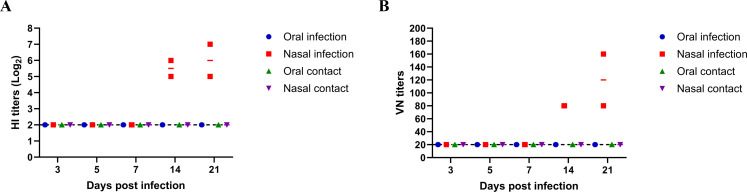
HI and neutralization titers of infected and contact calves. (A) HI titers and (B) virus neutralization titers of infected and contact calves of both intranasal and oral infection groups on different days post-infection.

The remaining two intranasally infected calves (#166 and #4074) seroconverted, with hemagglutination inhibition (HI) antibody titers against TX98 on 14 and 21 days post-infection reaching 32–128. In contrast, two orally infected calves (#164 and #168) did not seroconvert on either day (Fig. 5). Furthermore, neutralization antibodies against TX98, with titers ranging from 80 to 160, were detected in both intranasally inoculated animals on both 14 and 21 days post-infection. These results reveal that the swine TX98 H3N2 can infect calves and induce seroconversion, although no viral RNA and antigens were detected in the tissues of infected animals.

### Virus transmission among calves

To determine virus transmission, two naive animals were co-housed with four infected calves at day 2 post-infection in both infected groups. Contact calves in either the nasal or oral infection groups did not show any obvious clinical signs or fever. All swab samples collected from the contact calves, including nasal, oropharyngeal, and rectal swabs, were negative, and seroconversion was not detected. All results indicate that the TX98 H3N2 virus did not transmit among calves.

## DISCUSSION

Bovine influenza outbreaks caused by H5N1 HPAIV in dairy herds have caused significant economic loss to the U.S. bovine industry and raised attention regarding infections with other subtypes of IAVs in cattle and their potential public health risks. Although IAV infections in cattle have been reported, and human or swine IAVs have been isolated from cattle (18–20), the pathogenicity, dynamics, and tissue tropism of IAVs in cattle remain unclear. In this study, we first showed that primary respiratory cells and immortalized mammary gland cells from beef cattle are susceptible to infection with different subtypes of IAVs, including avian H5N1 and H9N2 and swine H3N2 viruses, but the avian H5N1 virus replicates in bovine cells more effectively than both avian H9N2 and swine H3N2 viruses. This suggests that other bovine species, such as beef cattle, are also susceptible to infections with different IAVs, despite current HPAIV H5N1 outbreaks in the United States being reported only in dairy cows and that variable susceptibility of the same cell line to different subtypes of IAVs might be correlated to virus tissue tropism and pathogenicity. Furthermore, we demonstrated that the swine H3N2 TX98 virus could infect and replicate in calves through intranasal infection, but not via oral infection, with no obvious clinical signs. Virus shedding in nasal swabs and seroconversion were detected in intranasally infected animals, while no evidence of virus infection was found in the orally infected calves, despite using a threefold higher virus dose for oral infection. This is likely due to the cattle gastrointestinal tract’s microenvironment being unsuitable for virus infection and replication despite the presence of sialic acid receptors (21). The TX98 H3N2 virus did not replicate extensively or transmit in calves, as no viral RNA or antigen was detected in tissues of intranasally infected animals necropsied on day 5 post-infection, and no virus infection (viral RNA and seroconversion) was detected in sentinel animals. This aligns with the field situation, as HI titers against TX98 H3N2 virus were detected in cattle herds, although no related disease had been reported based on our recent and previous studies (12, 22). Interestingly, minimal lung lesions consistent with IAV infection were observed in the right cranial and cardiac lung lobes of three necropsied calves (#175, #162, and #4073), but viral RNA and antigens could not be detected. This might be due to an inappropriate time window for detection and low viral abundance in these calves. Histological alterations in these animals were not consistent with bacterial bronchopneumonia, despite detecting *Staphylococcus aureus* in cattle #175 and *Streptomyces albidoflavus* in calf #174 by using aerobic culture (Table 3). In addition, deep sequencing of lung tissues did not identify pathogens associated with these lesions. The role of IAV infection in the development of these lesions could not be defined by IHC and virus detection. Notably, transient viremia was detected in only two intranasally infected calves on day 7 post-infection, and four amino acid mutations were identified in PA, NP, and NA proteins based on deep-sequencing results from these two serum samples. However, no identical mutations were found in both animals, and whether these mutations will promote virus adaptation and replication in cattle remains unknown and needs to be investigated. Although viremia was not found in experimentally infected cattle with H5N1 HPAIV (16, 17), viral RNA has been detected in serum or whole blood samples collected from dairy cattle on the H5N1 HPAIV outbreak farms by RT-qPCR assay (3). Additionally, the virus was detected in blood samples collected from mice, but not from ferrets that were intranasally infected with either an H5N1 HPAIV cattle isolate (A/dairy cattle/New Mexico/A240920343-93/2024) or an early human isolate (A/Vietnam/1203/2004) (23). Virus was also not detected in blood samples from mice and ferrets that were intranasally infected with a human H1N1 (A/Isumi/UT-KK001-01/2018) virus (23). These results suggest that viremia is likely transient and cleared rapidly in cattle, and whether virus infection induces viremia is dependent on the infected species, infection routes, and the IAV strain used. Further studies are needed to better understand the infection and replication of different IAVs in cattle.

**TABLE 3:** Results of swab, serum, and tissue samples tested by aerobic and anaerobic bacterial isolation, HI assay, and RT-qPCR^*a*^

Group	Calf		Nasal swab (0 dpi)						Serum (0 dpi)		Lung tissue (5 dpi)				
	Type	No.	Bacteria	IBRV	PIV3	BRSV	BVDV	Influenza D virus (IDV)	IAV (rgH5N1/PR8)	IAV (H3N2)	IBRV	PIV3	BRSV	BVDV	IDV
1 (nasal infection)	Infected	#175	*S. aureus*	−	−	−	−	−	−	−	−	−	−	−	−
		#4073	−	−	−	−	−	−	−	−	−	−	−	−	−
		#166	−	−	−	−	−	−	−	−	N/A	N/A	N/A	N/A	N/A
		#4074	−	−	−	−	−	−	−	−	N/A	N/A	N/A	N/A	N/A
	Contact	#176	−	−	−	−	−	−	−	−	N/A	N/A	N/A	N/A	N/A
		#4075	−	−	−	−	−	−	−	−	N/A	N/A	N/A	N/A	N/A
2 (oral infection)	Infected	#165	−	−	−	−	−	−	−	−	−	−	−	−	−
		#162	−	−	−	−	−	−	−	−	−	−	−	−	−
		#164	−	−	−	−	−	−	−	−	N/A	N/A	N/A	N/A	N/A
		#168	−	−	−	−	−	−	−	−	N/A	N/A	N/A	N/A	N/A
	Contact	#161	−	−	−	−	−	−	−	−	N/A	N/A	N/A	N/A	N/A
		#174	*S. albidoflavus* *S*. *amylolyticus*	−	−	−	−	−	−	−	N/A	N/A	N/A	N/A	N/A

^*a*^ “−,” negative; N/A, not collected.

Human H3N2 IAVs have been isolated from cattle with respiratory diseases (14, 19), indicating that they are likely contributing to disease. A previous study confirmed that the bovine virus A/cal/Duschanbe/55/71 caused an influenza-like illness and could be detected for 7 days in calves, while three human H3N2 Hong Kong influenza isolates failed to cause disease in inoculated calves (14). Our results and previously published studies indicate that human and swine H3N2 IAVs can infect cattle, but they do not replicate extensively or produce disease in experimentally inoculated calves, consistent with findings using an Equine/KY/94 (H3N8) virus to infect calves (12). Besides H3N2, the H1N1 virus has been found to infect cattle based on serological and experimental studies (11, 20, 24). Intranasal inoculation of calves with the A/sw/IL/75 (H1N1) virus resulted in respiratory disease with virus shedding, contact transmission to sentinel animals, and seroconversion (13). Previous studies also revealed that the ancestral human A/PR8 H1N1 induced productive intramammary infection and replication in experimentally inoculated dairy cows (25, 26), similar to findings from experimental infections with currently circulating H5N1 HPAIVs (16, 17). It is noticed that intranasal inoculation of cattle with H5N1 HPAIV did not induce obvious clinical diseases. One study showed that calves oronasally inoculated with either the U.S. HPAIV H5N1 B3.13 or EU H5N1 wild bird isolate resulted only in moderate nasal replication and shedding, with no severe clinical signs and transmission to sentinel calves (17). These results are similar to our results of calves intranasally infected with a swine H3N2 virus in this present study. Another study revealed mild clinical disease in heifers infected with H5N1 HPAIV by an aerosol respiratory route, with infection verified by virus detection, lesions, and seroconversion (16). All findings suggest that cattle clinical diseases are associated with infected IAV subtypes or strains, infection routes, and virus adaptation as well as potential coinfection with other pathogens. Further studies are needed to investigate whether other subtypes of IAVs can infect and cause disease in cattle.

To date, human- and swine-origin H3N2 and H1N1, as well as HPAIV H5N1 IAVs, have been documented to infect cattle, indicating that co-infection in some individual cattle with two or more IAVs might occur. Indeed, human H3N2 and H1N1 antibodies were detected in cattle herds in Northern Ireland and the United Kingdom that showed outbreaks of respiratory disease, milk drop syndrome, or diarrhea (9, 24, 27). Our recent retrospective serological studies found that some individual cattle were double or triple infected by human seasonal and swine IAVs (22). Nevertheless, IAVs from different species, including human and swine H1 and H3 viruses as well as HPAIV H5N1, can infect cattle, which has raised significant concern that reassortment might occur in cattle to generate novel reassortant H5 viruses with internal genes from human seasonal or North American triple reassortant swine IAVs. These novel reassortant viruses could more readily adapt to humans and other species and potentially cause the next pandemic if they gain human-to-human transmission. Therefore, it is necessary to perform systemic surveillance to monitor the epidemiology of IAVs in cattle to prevent potential further adaptation and pandemics in humans.

## MATERIALS AND METHODS

### Virus and cells

The viruses H3N2 A/Swine/Texas/4199-2/1998 (TX98), H9N2 A/Quail/Hong Kong/G1/1997 (HK97), H1N1 A/PR8/34 (PR8), and H5N1 rg-A/American wigeon/South Carolina/22-000345-001/2021 (rgH5N1/PR8) were used in this study. The clade 2.3.4.4b rgH5N1/PR8 virus is a reverse genetics-derived virus that contains six internal genes from the PR8 H1N1 virus and surface NA and modified HA genes from the clade 2.3.4.4b A/American wigeon/South Carolina/22-000345-001/2021 (H5N1) virus (22). The modified HA gene has only one basic amino acid at the HA cleavage site (R**↓**GLF), and its protein sequence shows 99.65% identity with two amino acid differences (L131Q and T211I) when compared to that of the bovine isolate clade 2.3.4.4b A/cattle/Texas/56283/2024 (H5N1) virus. The rgH5N1/PR8 NA protein shows 98.72% identity to the NA of the A/cattle/Texas/56283/2024 (H5N1) virus with amino acid differences I67V, S71N, E259D, M269L, I321V, and P339S. The HK97 H9N2 was selected in this study because the HK97-like virus has shown the ability to cross species barriers to infect humans (28) and pigs (29). TX98, PR8, and HK97 were generated by reverse genetics and amplified using Madin-Darby canine kidney cells, and rgH5N1/PR8 was amplified in SPF embryonated chicken eggs. All these viruses were titrated on MDCK cells. The TX98 H3N2 was used to inoculate calves as it had been shown to infect cattle based on our and other previous serological studies (12, 22). MDCK cells were maintained in minimum essential medium (Corning, Manassas, VA, USA), supplemented with 10% fetal bovine serum (FBS; Gibco, Waltham, MA, USA) and 1% antibiotic-antimycotic solution (Gibco, Waltham, MA, USA). The infection medium used for virus cultivation was supplemented with 0.3% bovine serum albumin (Sigma, St. Louis, MO, USA), 1% minimum essential medium vitamin solution (Gibco, Waltham, MA, USA), 1% L-glutamine (Gibco, Brazil), and 1% antibiotic-antimycotic solution in addition to 1 µg/mL of TPCK-treated trypsin (Worthington-Biochem, Lakewood, NJ, USA).

### Establishment of primary epithelial cell cultures from the bovine respiratory tract and mammary gland and infection of established cells with different IAVs

Nasal turbinate, tracheal, and mammary gland tissues were collected from one beef cattle slaughtered at the University of Missouri meat science laboratory. Tissues were collected immediately after slaughter in DMEM, washed with 1× PBS, cut into small pieces, and incubated with collagenase enzyme at 37°C for 1.5 h. The cells were then strained through 70 µm cell strainers, followed by centrifuging at 500 × *g* for 5 min. The cell pellet was washed and seeded on collagen-coated flasks. Airway epithelial cells were incubated at 37°C with 5% CO_2_ and maintained using bronchia/trachea epithelial cell basal medium (Cell Applications, Inc.) supplemented with 10% FBS (Gibco, Waltham, MA, USA). Mammary epithelial cells were cultured in DMEM (Corning, Manassas, VA, USA) supplemented with 10% FBS. All media for primary cell cultures were applied with 100 U/mL penicillin and 100  µg/mL streptomycin. Non-adherent cells were washed off the flask after 24 h of culturing. Fibroblasts were removed by treating the cells with 0.03% trypsin every 3 days. Developed primary cells were stored in liquid nitrogen after two passages for future studies. Isolated mammary gland cells were further immortalized with the HT Lenti-SV40T-PURO Immortalization Kit (Creative Bioarray, CIK-HT035) according to the manufacturer’s instructions. The immortalized cells were stored in liquid nitrogen after two passages for future studies.

Specific antibody staining was performed to validate the established four bovine cell lines. Primary nasal turbinate and tracheal epithelial cells, as well as immortalized mammary gland epithelial cells, were stained with the Alexa Fluor 488-conjugated mouse anti-pan cytokeratin monoclonal antibody (AE1/AE3) (1:500, Fisher, 53-9003-82), while the immortalized mammary gland fibroblasts were stained with the Alexa Fluor 488-conjugated mouse SMA monoclonal antibody (1A4), (1:500, Fisher, 53-9760-80). Each cell line was stained with a Mouse IgG isotype (1:500, Santa Cruz, sc-2025), followed by the Alexa Fluor 488-conjugated goat anti-mouse secondary antibody (1:1,000, Fisher, A-11001) as controls. For cell nuclei staining, 4′,6-diamidino-2-phenylindole, dihydrochloride (Fisher, 62247) was used.

A monolayer of bovine primary respiratory cells and immortalized mammary gland cells in 6-well plates was infected with the rgH5N1/PR8, TX98 H3N2, HK97 H3N2, or PR8 H1N1 virus at a multiplicity of infection (MOI) of 1 in the DMEM with 1 µg/mL TPCK-treated trypsin. After 1 h of adsorption at 37°C, the virus inoculum was removed and then incubated with DMEM without supplements for 24 h. Cells were then fixed with cold methanol and blocked with 5% skim milk, followed by staining with an NP monoclonal antibody (HB65, ATCC H16-L10-4R5) and Alexa Fluor 488-conjugated goat anti-mouse secondary antibody (A-11001, Eugene, Oregon). To quantify infected cells, images from 12 random fields of three separate biological repeats were acquired using the EVOS M5000 imaging system under consistent settings. Images were processed and quantified in batches using ImageJ software (30). Briefly, images were converted to 8 bit, and a threshold of 20–255 was applied to exclude background noise and capture all fluorescence signals. All images were analyzed using consistent settings, and mean values from three biological replicates were compared.

To determine virus replication, a monolayer of bovine primary respiratory cells and immortalized mammary gland cells in 12-well plates was infected with the rgH5N1/PR8, TX98 H3N2, or HK97 H3N2 virus at an MOI of 0.1 in the DMEM with 1 µg/mL TPCK-treated trypsin. The supernatants of each virus-infected cell were collected at 12 and 36 h post-infection and then titrated on MDCK cells. The 50% tissue culture infective dose (TCID_50_)/mL was calculated using the method of Reed and Muench.

### Calf infection

Twelve Holstein calves (1.5–2.5 months of age), consisting of six males and six females, were purchased from the University of Missouri dairy farm and transported to the NextGen Center for Influenza and Emerging Infectious Diseases vivarium for a 5 day acclimation. Each calf was fed twice (morning and afternoon) with a bottle of milk [12 ounces of MFA milk replacer (Millersburg, MO, USA) in 2 quarts of warm water] daily and provided MFA sweet feed (Millersburg, MO, USA) and water *ad libitum* all day. Upon arrival, blood and nasal swabs were collected and screened for current or recent IAV infection, various pathogens associated with bovine respiratory disease complex, and influenza D virus. Calves were negative for any active or recent IAV infections based on CDC influenza RT-qPCR targeting the M gene (31) and HI assay results testing for both TX98 and rgH5N1/PR8 viruses (Table 3).

Calves were divided into two groups: one group of four calves, including two males and two females (#175, #166, #4074, and #4073), was intranasally infected, and one group of four calves, including two males and two females (#165, #164, #168, and #162), was orally infected. Prior to infection, blood, nasal, oropharyngeal, and rectal swab samples were collected from each animal. Each calf in the nasal infection group was intranasally inoculated with 10⁸ TCID_50_ of TX98 on day 0, while each animal in the oral infection group was orally infected by feeding with milk-virus mixtures (containing 10⁸ TCID_50_ of TX98) for three successive days on days 0, 1, and 2 (once per day). Two naive calves, including one male and one female, were co-housed with infected animals at 2 days post-infection in intranasally (#176 and #4075) or orally (#161 and #174) infected groups to investigate virus transmission (Fig. 3A).

Clinical signs and fever (rectal temperature) were monitored daily. Nasal, oropharyngeal, and rectal swabs were collected from each calf daily until day 14 post-infection to monitor viral shedding. Blood samples were taken on 3, 5, 7, 14, and 21 dpi to determine viremia and seroconversion. Two infected animals, including one male and one female from each infection group, were sacrificed on day 5 post-infection to determine pathogenicity and virus replication (Fig. 3A). During necropsy, tissues were collected from each calf, including abomasum, kidney, liver, lung lobes, omasum, reticulum, rumen, spleen, thymus, trachea, nasal turbinate, brainstem, cerebellum, and various lymph nodes (ileocecal, inguinal, mandibular, mesenteric, parotid, popliteal, and retropharyngeal). The collected tissues were frozen for virological analysis or fixed in 10% neutral-buffered formalin for histopathological analysis.

### RT-qPCR and virus titration

Viral RNA load in tissue, serum, and swab samples was determined by a house-developed specific RT‐qPCR that targets the TX98 HA gene. RNA was extracted from swab supernatants, homogenized tissue samples, and serum samples using the Qiagen Kit (Germantown, MA, USA). Primers (Fw: 5′-gctgaggacatgggcaatggttg-3′; Re: 5′-ccggttgtttaatgcttcgtctctg-3′) and a probe (5′-(FAM)-ttgaccctatgcaggcattgtcac-(BHQ1)-3′) targeting the TX98 HA gene were used in a one-step RT-qPCR assay (Quantabio, Beverly, MA, USA). The RT-qPCR process includes a 10 min reverse transcription and a 1 min initial denaturation at 95°C, followed by 45 amplification cycles at 95°C for 10 s and 60°C for 40 s. Amplifications were conducted in a 20 μL reaction volume containing 5 μL of RNA, 0.125 µM of each primer, and 0.125 µM of probe. The cutoff value of this specific RT-qPCR is 40, and it is capable of detecting 0.01 TCID_50_/mL of the TX98.

RT-qPCR-positive samples were further processed for virus titration on MDCK cells as described previously (32). Briefly, positive samples, such as nasal swab supernatants, were serially diluted with infection medium from 10⁻¹ to 10⁻^8^ and then inoculated onto MDCK cell monolayers in 96-well plates. Cytopathic effects on inoculated cells were observed daily. After 3–4 days of incubation, cells were fixed with cold methanol and stained with an anti-IAV NP monoclonal antibody (HB65, ATCC H16-L10-4R5). The TCID_50_/mL was calculated using the method of Reed and Muench.

### Hemagglutination inhibition and microneutralization assays

The collected serum samples were mixed with Receptor Destroying Enzyme II (Denka, Tokyo, Japan) at a 1:3 ratio, and then the mixture was incubated at 37°C for 18–20 h, followed by heating at 56°C for 30–60 minutes for the HI assay. The treated serum sample was serially diluted from 2^−2^ to 2^−10^ in PBS and incubated with 4 hemagglutinating units (HAU) of TX98 at room temperature for 30 minutes. Then, 0.5% chicken red blood cells (RBCs) were added and incubated at room temperatue for 30 minutes. PBS incubated with and without 4 HAU of TX98 served as positive and negative controls, respectively. The HI titer represents the highest serum dilution that prevents virus-induced hemagglutination of RBCs.

For the microneutralization assay, cattle serum samples were inactivated at 56°C for 30 minutes. The serum sample was twofold serially diluted from 1:20 to 1:2,560 in infection medium and incubated with 100 TCID_50_ of TX98 at 37°C for 1 h. The mixture was then transferred to confluent MDCK cell monolayers on 96-well plates and incubated at 37°C with 5% CO_2_. Cytopathic effects were observed daily, and after 3–4 days of incubation, cells were fixed and stained with an anti-IAV NP monoclonal antibody (HB65, ATCC H16-L10-4R5). The neutralizing antibody titer represents the highest dilution that completely inhibits virus replication.

### Microscopic pathology and influenza A virus-specific immunohistochemistry

Post-mortem examinations were performed on day 5 post-infection, and an experienced veterinarian recorded the percentage of gross lung lesions. Trachea, lung, bronchi, and nasal turbinate samples were processed with standard histological techniques and stained with hematoxylin and eosin (H&E). Remaining tissue samples that were positive by specific RT-qPCR were further processed for H&E staining. A board-certified veterinary pathologist was blinded to examine histopathological changes as described previously (17). Immunohistochemistry analysis was conducted to detect influenza NP antigen using a rabbit polyclonal antibody (Fisher, Rockford, IL, USA), with a rabbit IgG isotype (Fisher, Rockford, IL, USA) used as a negative control.

### Deep sequencing and data analysis

One milliliter of lung homogenate or serum was centrifuged at 8,000 × *g* for 2 minutes, and 400 μL of the resulting supernatant was used for RNA extraction. This process included optional Proteinase K and DNase I digestion steps, as specified in the ZYMO Quick-DNA/RNA Viral Kit protocol (Zymo, Irvine, CA, USA). RNA concentrations in the four lung samples of calves necropsied at 5 dpi ranged from 25 to 88 ng/μL, measured with a Qubit 4 fluorometer using the Qubit RNA HS Kit (ThermoFisher, Waltham, MA, USA). RNA concentrations of eight serum samples collected from infected and contact calves in the intranasal infection group at 5 and 7 dpi were below detection limits. For library preparation, 8 μL of serum RNA or 250 ng of lung RNA was processed with the QIAseq Single Cell RNA Library Kit (Qiagen, Waltham, MA, USA). A ribosomal RNA (rRNA) depletion step was included, using the QIAseq FastSelect–rRNA HMR Kit (Qiagen, Waltham, MA, USA), following the manufacturer’s instructions. The 12 libraries were pooled in equimolar amounts and sequenced using a MiSeq 150 × 2 bp kit.

Bioinformatic analysis was performed using CLC Genomics Workbench 24 (Qiagen, Waltham, MA, USA). Briefly, raw reads were assessed for sequencing quality, and adapter and low-quality bases were trimmed. Reads from cattle were then removed, and the remaining reads were mapped against the reference genome of influenza A/swine/Texas/4199-2/1998 (H3N2) virus (NCBI Genome Assembly ASM3817019v1).

### Statistical analysis

The significance of differences between groups and virus titers was analyzed by using a one-way ANOVA followed by Tukey’s multiple comparison, a two-way ANOVA in GraphPad Prism 10 software (GraphPad), or a Student’s *t* test. *P* < 0.05 was determined to be statistically significant (*P < 0.05, **P < 0.01, ***P < 0.001, and ****P < 0.0001). Error bars represent standard deviation (±SD).

## Data Availability

Data presented in this study are available upon request. Materials are available upon request through the filing of material transfer agreements.

## SUPPLEMENTAL MATERIAL

Supplemental tables mbio.03957-24-s0001.docx Tables S1 to S3.

Legends mbio.03957-24-s0002.docx Legends for Fig. S1 and S2.

Figure S1 mbio.03957-24-s0003.tif Morphology and antibody staining of established bovine primary respiratory cells and immortalized mammary gland cells.

Figure S2 mbio.03957-24-s0004.tif Hematoxylin and eosin-stained respiratory section of H3N2 virus-infected animals.

ASM does not own the copyrights to Supplemental Material that may be linked to, or accessed through, an article. The authors have granted ASM a non-exclusive, world-wide license to publish the Supplemental Material files. Please contact the corresponding author directly for reuse.
