# Supplementary material for: Outcomes of experimental infection of calves with swine influenza H3N2 virus
Source: mBio. 2025 Jun 12;16(7):e03957-24. doi: 10.1128/mbio.03957-24 (PMC12239592; doi:10.1128/mbio.03957-24)
Supplement: Supplemental tables — Tables S1 to S3. [file mbio.03957-24-s0001.docx]

**Supplemental Tables**

**Table S1: Results of Tissue samples tested by RT-qPCR**

|  | **5 dpi** | | | |
| --- | --- | --- | --- | --- |
|  | Group 1  (Nasal infection) | | Group 2  (Oral infection) | |
|  | #175 | #4073 | #165 | #162 |
| Abomasum | − | − | − | − |
| Kidney | − | − | − | − |
| Liver | − | − | − | − |
| Iieocecal LN | − | − | − | − |
| Inguinal LN | − | − | − | − |
| Mandibular LN | − | − | − | − |
| Parotid LN | − | − | − | − |
| Popliteal LN | − | − | − | − |
| Retropharyngeal LN | − | − | − | − |
| Lung | − | − | − | − |
| Omasum | − | − | − | − |
| Reticulum | − | − | − | − |
| Rumen | − | − | − | − |
| Spleen | − | − | − | − |
| Thymus | − | − | − | − |
| Trachea | − | − | − | − |
| Turbinate | − | − | − | − |
| Brainstem | − | − | − | − |
| Cerebellum | − | − | − | − |

−, Ct ≥ 40.

**Table S2: Deep sequencing results of lung and serum samples**

|  | **sample ID** | sample type | raw reads | reads after trimming | cattle reads | ASM3817019 reads |
| --- | --- | --- | --- | --- | --- | --- |
| Oral infection group (infected) | 162 | lung | 2,309,862 | 2,305,979 | 570,886 | 0 |
|  | 165 | lung | 2,104,638 | 2,101,698 | 672,882 | 0 |
| Nasal infection group (infected) | 175 | lung | 1,906,706 | 1,902,092 | 625,584 | 0 |
|  | 4073 | lung | 1,910,980 | 1,904,092 | 714,990 | 0 |
|  | 166 | serum, 5 dpi | 3,653,410 | 3,653,065 | 1,581,978 | 0 |
|  | 166 | serum, 7 dpi | 4,438,582 | 4,434,866 | 1,730,646 | 126 |
|  | 4074 | serum, 5 dpi | 4,419,732 | 4,418,471 | 902,018 | 0 |
|  | 4074 | serum, 7 dpi | 4,049,762 | 4,047,993 | 1,887,414 | 304 |
| Nasal infection group (contact) | 176 | serum, 5 dpi | 4,502,190 | 4,501,747 | 34,374 | 0 |
|  | 176 | serum, 7 dpi | 4,582,298 | 4,579,434 | 1,215,368 | 0 |
|  | 4075 | serum, 5 dpi | 3,852,274 | 3,850,773 | 1,925,136 | 0 |
|  | 4075 | serum, 7 dpi | 4,623,358 | 4,621,971 | 1,474,048 | 0 |

**Table S3: Viral RNA fragments detected in serum samples on 7 dpi**

| **Sample** | **Segment** | | | | | | | | | | | | | |  |
| --- | --- | --- | --- | --- | --- | --- | --- | --- | --- | --- | --- | --- | --- | --- | --- |
|  | **PB2** | **PB1** | **PA** | | **HA** | | **NP** | | | **NA** | | **M** | | **NS** | |
| #166 | 1295-1428 (C1300A/R427R)* |  | 1530-1599 | | 361-465 | |  | | |  | |  | |  | |
|  |  |  | 1629-1909  (G1740C/G578A) | | 493-802 | |  | | |  | |  | |  | |
| #4074 | 505-591 | 928-1202 | 904-1284 | | 197-395 | | 694-852 (G804C/E260Q) (T835G/L270R) | | | 155-246  (T213A/V66E) | | 177-510 | |  | |
|  | 711-1189 | 1211-1322 | |  | | 495-1015 | |  | 262-412 | | 547-873 | |  | |  |
|  | 1224-1414 | 1355-1952 | |  | | 1348-1462 | |  | 800-1176 | |  | |  | |  |
|  |  | 1954-2129 | |  | | 1470-1703 | |  |  | |  | |  | |  |

* Mutations detected in each segment (mutations at nucleotide level/mutations at amino acid level)
