# Supplementary material for: Outcomes of experimental infection of calves with swine influenza H3N2 virus
Source: mBio. 2025 Jun 12;16(7):e03957-24. doi: 10.1128/mbio.03957-24 (PMC12239592; doi:10.1128/mbio.03957-24)
Supplement: Legends — for Fig. S1 and S2. [file mbio.03957-24-s0002.docx]

**Legends for Supplemental Figures**

**Fig. S1. Morphology and antibody staining of established bovine primary respiratory cells and immortalized mammary gland cells.** A) Morphology of four bovine cell cultures and MDCK cells. B) Four bovine cell lines were stained with the Alexa Fluor™ 488-conjugated mouse anti-cytokeratin or -SMA monoclonal antibody. Each cell line was stained with a Mouse IgG antibody, followed by the Alexa Fluor™ 488-conjugated goat anti-mouse secondary antibody as controls. DAPI was used to stain the cell nuclei.

**Fig. S2. Hematoxylin and eosin- stained respiratory section of H3N2 virus infected animals.** A) Trachea (#165); B) Nasal Turbinate (#165); C) Lung (#175). Animals #162, #165, #175, and #4073 had inflammatory cells (macrophages, lymphocytes, neutrophils, eosinophils) in mucosa and submucosa of both trachea and nasal turbinate. Animal #175 had a focally extensive area of inflammation (neutrophils, macrophages, lymphocytes) in lumen of bronchioles and alveoli. Inflammation (neutrophils, macrophages).
