# Supplementary figures and images for: Outcomes of experimental infection of calves with swine influenza H3N2 virus
Source: mBio. 2025 Jun 12;16(7):e03957-24. doi: 10.1128/mbio.03957-24 (PMC12239592; doi:10.1128/mbio.03957-24)

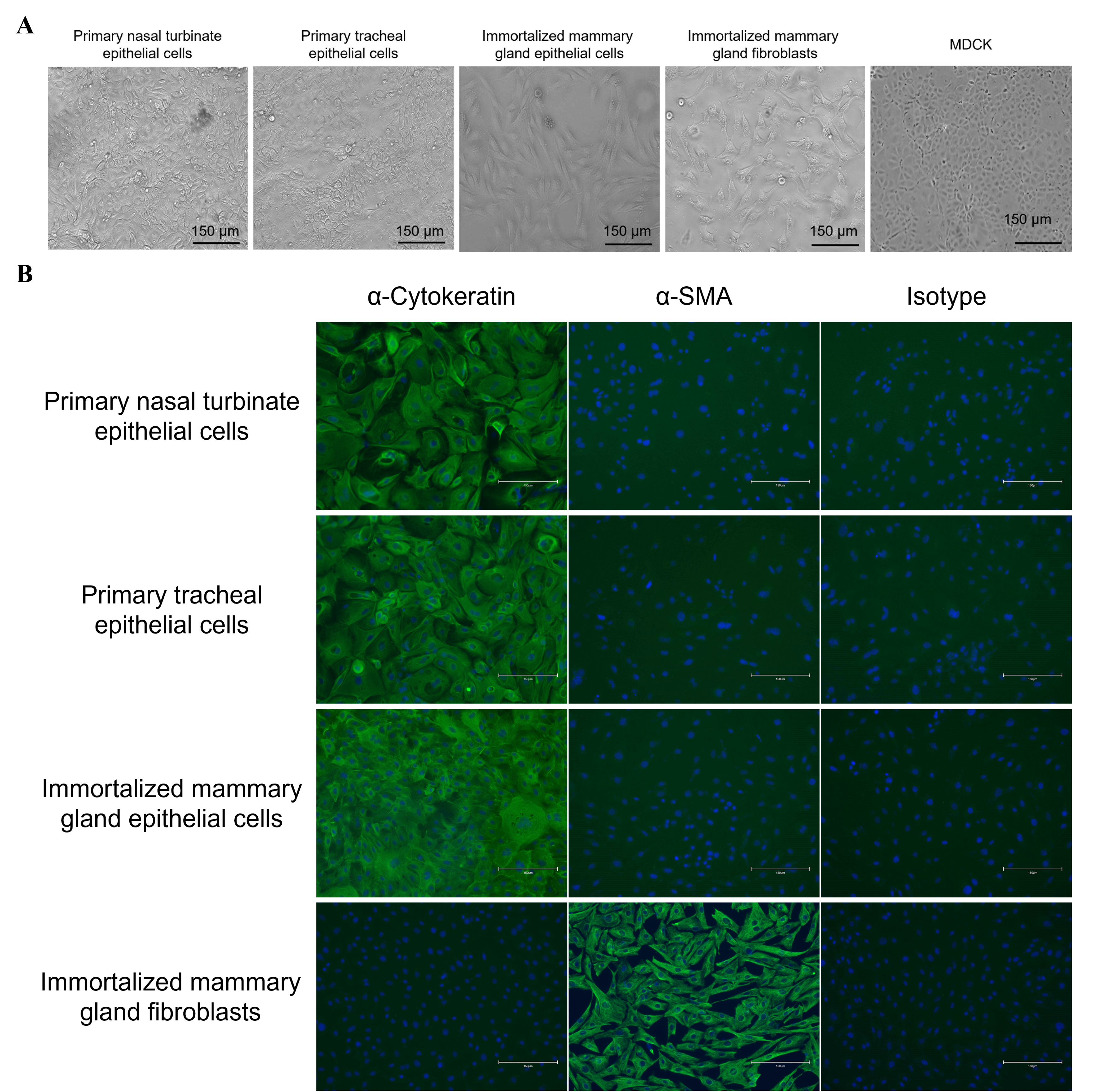

Supplement: Figure S1 — Morphology and antibody staining of established bovine primary respiratory cells and immortalized mammary gland cells. [file mbio.03957-24-s0003.tif]
